# A Conundrum of Colliding Conditions: A Histopathological Case Report of Chiari Type III with Complete Spina Bifida Aperta

**DOI:** 10.3390/reports8040202

**Published:** 2025-10-12

**Authors:** George Stoyanov, Ivaylo Balabanov, Svetoslava Zhivkova, Hristo Popov

**Affiliations:** 1 Department of Pathology, Multiprofile Hospital for Active Treatment, Vasil Aprilov 61 Str., 9700 Shumen, Bulgaria; 2 Department of Obstetrics and Gynecology, Multiprofile Hospital for Active Treatment, 9700 Shumen, Bulgaria; 3 Department of General and Clinical Pathology, Forensic Medicine and Deontology, Faculty of Medicine, Medical University–Varna, 9000 Varna, Bulgaria

**Keywords:** spina bifida, Chiari malformation, Arnold-Chiari, Chiari type III, cervicothoracic meningomyelocele, meningomyelocele

## Abstract

**Background and Clinical Significance**: Spina bifida in the cervical region is closely associated with Chiari malformation, which is an amalgamation of terminology for separate conditions with similar pathophysiological mechanisms and progression from one another. Chiari malformations are associated with varying degrees of dilation of the foramen magnum or lack of fusion of the occipital bone with syringomyelia, herniation of the cerebellum, occipitocele and occipitomyelocele; **Case Presentation**: A previously healthy 23-year-old primigravida presented to our institution due to fetal demise in the third lunar month, established on routine outpatient maternal consultation. Point-of-care ultrasound revealed an amniotic sac measuring 3 cm in diameter and containing a single fetus, without cardiac function. Due to these, the patient was scheduled for pregnancy termination, during which the cervix was noted to be spontaneously dilated and abrasion accomplished complete evacuation of the amniotic sac, without its rupture. Upon sectioning of the amniotic sac, a fetus, measuring 2.5 cm in length, was noted, with a significant cuffing of the occipital and cervical paraspinal region. Histology revealed fetal structures with an adequate maturation index for its gestational age, but it presented with a pronounced meningoencephalomyelocele in the cervical and thoracic regions, characterized by the complete absence of vertebral arches and spinous processes from the atlanto-occipital to the sacral region; **Conclusions**: In the present case, not only is a significant and complex form of Chiari type III reported, but the condition is also associated with spina bifida aperta in all spinal regions, leading to meningoencephalomyelocele, incompatible with life.

## 1. Introduction and Clinical Significance

Spina bifida is a congenital anomaly characterized by the failure of the vertebral arches to fuse during fetal development [1]. The condition is relatively common overall, with epidemiological reports indicating an incidence of 0.13% of all births, the median for Africa (the lowest reported rate), or a global incidence of up to approximately 40 cases per 1000 live births. While generally depicted under the umbrella term of spina bifida and similar in their pathophysiology and development, several conditions fall under this generalization [2,3,4]. The two main types of spina bifida are closed (oculta) and open (aperta) [1]. In Spina bifida oculta, there is a failure of neural tube closure in which the vertebral arches do not fuse, but this does not affect the spinal cord as the fusion failure is too small to allow for herniation [5,6]. This form is probably globally underreported as it rarely has significant clinical signs and is often an incidental finding later on in life [5,6]. Spina bifida aperta is a more severe form wherein the fusion defect is larger, and this allows protrusion and herniation of either the meninges alone (meningocele) or the meninges and the spinal cord altogether (meningomyelocele) [1]. In this form, due to the herniation and compression, first of all, there are both definitive ultrasound findings in utero, clearly visible herniation and almost always a significant degeneration of the neural tissues, leading to denervation and loss of mobility and sensation beneath the affected segment postpartum [1]. Changes typically start to develop prior to the second lunar month, as normally not only vertebral arch fusion but also ossification are already apparent in the third one [1].

The incidence of spina bifida aperta with meningomyelocele is estimated to be 1 in 1000 live births [1]. The most commonly affected areas of the spinal cord are the lumbar, followed by the sacral segments, with the thoracic and especially the cervical regions being exceptionally rare [7,8,9].

Spina bifida in the cervical region is closely associated with Chiari malformation, which is an amalgamation of terminology for separate conditions with similar pathophysiological mechanisms and progression from one another [10]. Chiari malformations are associated with varying degrees of dilation of the foramen magnum or lack of fusion of the occipital bone with siringomielia, herniation of the cerebellum, occipitocele and occipitomyelocele [10]. The designated Achold-Chiary malformations, Chiari type II and type III, are closely related in their morphology and mechanisms to cervical spina bifida [10]. All types of Chiari malformation exhibit at least minimal to prevalent clinical symptoms, with some, such as type 3.5, which has only one reported case of occipitocervical encephalocele with communication to the stomach, being incompatible with life [11].

## 2. Case Presentation

A previously healthy 23-year-old primigravida presented to our institution due to fetal demise established on routine outpatient maternal consultation. Outpatient medical documentation and the patient reported the last previous menstruation that indicated that pregnancy had just entered the third lunar month. No maternal or other risk factors were reported, such as previous or family history of fetal demise or birth defects, consanguinity or medications, apart from lack of folic acid prophylaxis.

Point-of-care ultrasound revealed an amniotic sac measuring 3 cm in diameter and containing a single fetus, without cardiac function. Due to these, the patient was scheduled for pregnancy termination, during which the cervix was noted to be spontaneously dilated and abrasion accomplished complete evacuation of the amniotic sac, without its rupture. The post-intervention period was uneventful.

**Figure 1:**
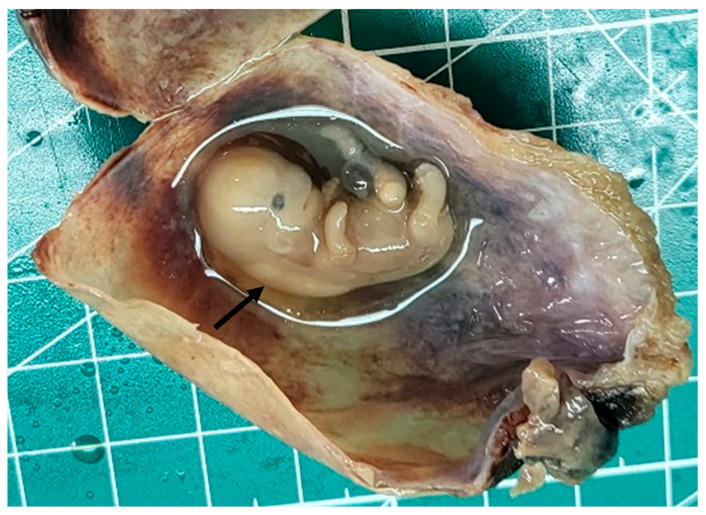
Gross view. Suboccipital and perispinal cuffing (arrow).

The specimen from abrasion, containing the unruptured amniotic sac and placental fragment, was sent to pathology for evaluation. Upon sectioning of the amniotic sac, a fetus, measuring 2.5 cm in length, was noted, with a significant cuffing of the occipital and cervical paraspinal region (Figure 1).

**Figure 2:**
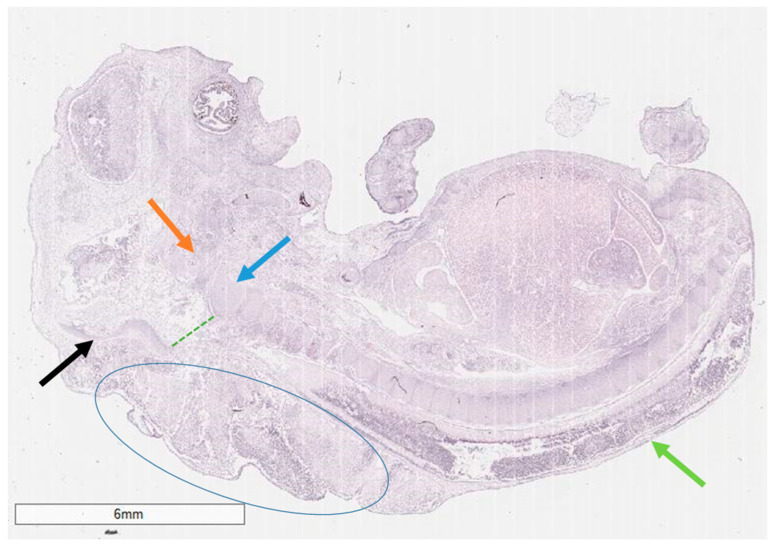
Histology of the fetus, macro-slide view. Foramen magnum (green punctuated line), occipital bone (black arrow), clivus (orange arrow), second cervical vertebrae body (blue arrow), complete absence of vertebral arches and spinous processes in all vertebral regions with subcutaneous location of the spinal cord (green arrow), subcutaneous herniated meninges and brain matter (blue ellipse); Hematoxilin and eosin stain.

**Figure 3:**
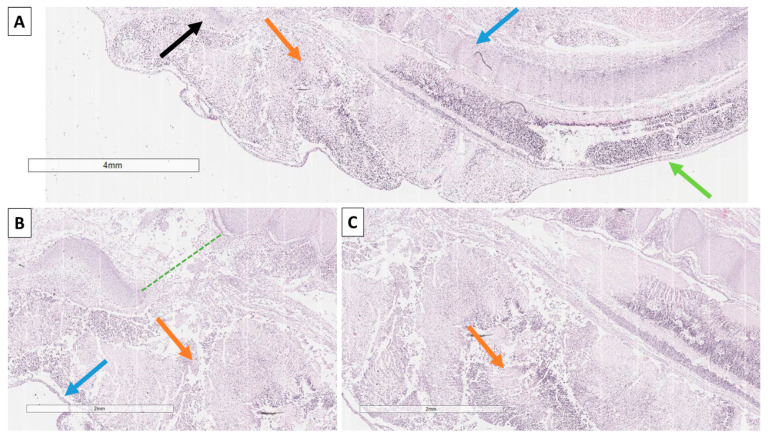
Histology of the fetus. (**A**) occipital bone (black arrow), herniated meninges and brain matter (orange arrow), spinal bodies (blue arrow), complete absence of vertebral arches and spinous processes in all vertebral regions with subcutaneous location of the spinal cord (green arrow); Hematoxylin and eosin stain, original magnification 15×; (**B**) foramen magnum (green punctuated line), cervical skin (blue arrow) and herniated meninges and brain matter (orange arrow), Hematoxylin and eosin stain, original magnification 20×; (**C**) cerebellar structures (orange arrow) within the subcutaneous herniation, Hematoxylin and eosin stain, original magnification 20×.

Histology revealed an amniotic sac with pronounced dyscirculatory changes, chorionic villi with preserved morphological characteristics and edema. The fetus itself had fetal structures with an adequate maturation index for its gestational age, but it presented with a pronounced meningoencephalomyelocele in the cervical and thoracic regions, characterized by the complete absence of vertebral arches and spinous processes from the atlanto-occipital to the sacral region (Figure 2 and Figure 3). The cause of the miscarriage was established as a severe malformation of fetal development incompatible with life, a complex Chiari type III-like malformation with spina bifida aperta in all spinal regions.

## 3. Discussion

As underlined by the presented case, Chiari type III malformations are rarely compatible with life, and for those cases that are compatible, they are often accompanied by significant neurological deficit [12,13]. In our case, as illustrated by the figures, the fetus has not only a complex type III Chiari malformation but also spina bifida in all spinal segments, underscoring the complex and interconnected etiopathogenesis of these conditions, which may represent an extreme spectrum of variances [14,15]. Although often associated with maternal folic acid deficit, the development of these conditions is often complex and includes both maternal deficits and genetic factors [15,16,17]. Despite these and probably due to the overall rarity of Chiari malformations, no recurrent cases have been reported in subsequent pregnancies.

Screening for both Chiari malformation and spina bifida during pregnancy follows the same steps, with folate level measurement, as they show the strongest statistical correlations and regular fetal ultrasound [17,18,19].

To date, there have been fewer than 100 cases of Chiari type III malformations reported [13,18,20]. In the reported instances, encephalocele occurs either in the occipital or cervical regions, with our case, to the best of our knowledge, being the only one with complete spina bifida of all spinal sections [13,18,20]. This displacement typically, but not always, leads to significant neurological and developmental deficits, although some cases report a relatively indolent clinical course, especially if surgical reconstruction is a viable option [13,20].

Unlike in spina bifida, where the exact mechanism of the anomaly’s development is well established, the mechanisms for Chiari type III are widely debated and often contradictory to one another. One proposed mechanism is that of a primary mesodermal defect leading to defective formation of the occipital bone, and hence, posterior fossa hypoplasia, with subsequent nervous system distention and herniation [18]. This mechanism would, however, only be viable in cases of occipitoencephalocele. A second proposed mechanism is that of a primary defective neural tube with cerebrospinal fluid leakage and improper distention of the cerebral ventricular system, which in itself leads to microcephaly and prolapse and herniation of the posterior fossa elements [18]. This mechanism would be more plausible in type II Chiari malformation, as well as type III with cervical encephalocele, such as in our case, but not in those with occipitoencephalocele.

In our view, the mechanisms are probably complex and synergic between the two most widely accepted and previously depicted ones. A defect in the occipital bone formation is likely to cause cerebrospinal fluid flow anomaly, leading to herniation of cerebral and cerebellar elements, as well as disorganization and hypoplasia of the posterior fossa. On this background, the location of the mesodermal defect itself is also responsible for the location of herniation—occipital or cervical.

As the mechanisms leading to the development of Chiari type malformations and spina bifida have significant structural and developmental stage overlap, in our view, it is most likely that the presented case represents an extreme scenario wherein both the neural tube and mesoderm develop synchronous anomalies. As such, likely the leading mechanism is that of severe cerebrospinal fluid leakage with pressure overload leading to a funneling effect. As such, it would explain both the variety of conditions with complete lack of vertebral arches in all spinal segments and why the herniations are most severe in the occipital and cervicothoracic regions of the fetus.

Despite the complex morphology of the case, no specific risk factors were established in the mother, apart from a lack of folic acid supplementation. Although regarded as a risk factor for spina bifida and as underlined in the previous section, folic acid deficiency is by no means the single causative agent [15,16,17]. As per the presented case, maternal folic acid deficit can be viewed at best as a triggering factor, unlocking a myriad of genetic and possibly potentiating other environmental factors involved in this complex dysmorphogentic condition [15,16,17].

## 4. Conclusions

Chiari malformations are rare and often associated with spina bifida. Despite some cases reporting an indolent clinical course, especially favorable if surgical reconstruction is viable and performed early, neurological and developmental deficits can be significant. In the present case, not only is a significant and complex form of Chiari type III reported, but the condition is also associated with spina bifida aperta in all spinal regions, leading to not only cervical but also cervico-thoracic encephalocele. As such, the condition was incompatible with life.

## Data Availability

The original contributions presented in this study are included in the article. Further inquiries can be directed to the corresponding author.
